# One case of iodine-125 therapy – A new minimally invasive treatment of intrahepatic cholangiocarcinoma

**DOI:** 10.1515/biol-2022-0473

**Published:** 2022-09-27

**Authors:** Xinju Chen, Xiaoqi Chen, Chuanlei Zhang, Xinting Wang, Changwei Yuan, He Yang, Lixia Yang

**Affiliations:** Department of Purchasing Center, The First Affiliated Hospital of Henan University of Chinese Medicine, No. 19, Renmin Road, Zhengzhou, Henan, 450000, P.R. China

**Keywords:** intrahepatic cholangiocarcinoma, transcatheter arterial chemoembolization, iodine-125

## Abstract

Intrahepatic cholangiocarcinoma (ICC) is the second most common primary liver cancer associated with a poor prognosis. ICC accounts for about 10% of primary liver malignancies but with increasing incidence in recent years. Recently, some studies suggested that minimally interventional therapy can be used in the treatment of ICC. However, there are few references on interventional therapy for the clinical treatment of ICC. Herein we reported a case of a 48-year-old man who suffered from ICC. The patient was diagnosed with ICC by computerized tomography scan and pathological biopsy. The patient was completely cured by minimally interventional therapy with iodine-125 seed implantation. These results provide an important reference for the treatment option of ICC.

## 1 Background

Intrahepatic cholangiocarcinoma (ICC) is a primary malignant tumor originating from secondary bile ducts and above the peripheral small bile ducts in the liver [1]. Due to its intrahepatic localization, early symptoms are not obvious, and most patients are diagnosed with advanced ICC [2]. Although in the past few years, there have been several reports pointing to the general value of liver resection in the management of ICC. However, there is still great uncertainty regarding the surgical management of large and advanced ICC [3]. Recently, many studies have shown that interventional therapy is a potential treatment method for ICC [4,5]. Iodine-125 brachytherapy is a technique that has been used recently to treat spine and bone metastases, as well as inoperable brain tumors and head and neck cancers [6,7,8,9]. As an interventional treatment, brachytherapy via iodine-125 seed implantation has developed over the past decades and has become an acceptable palliative treatment for patients with unresectable cholangiocarcinoma [10]. It has been reported that self-expandable metallic stent combined with catheter-loaded iodine-125 seeds brachytherapy significantly improves outcomes and could be a safe and effective treatment for advanced extrahepatic cholangiocarcinoma [11]. However, little research related to the iodine-125 brachytherapy for ICC have been reported till now. Herein we reported a case of 48-year-old man who suffered from ICC and completely cured by minimally interventional therapy with iodine-125 seed implantation. These results provide the important reference for treatment option of ICC.

### 1.1 Case presentation

**Figure 1:**
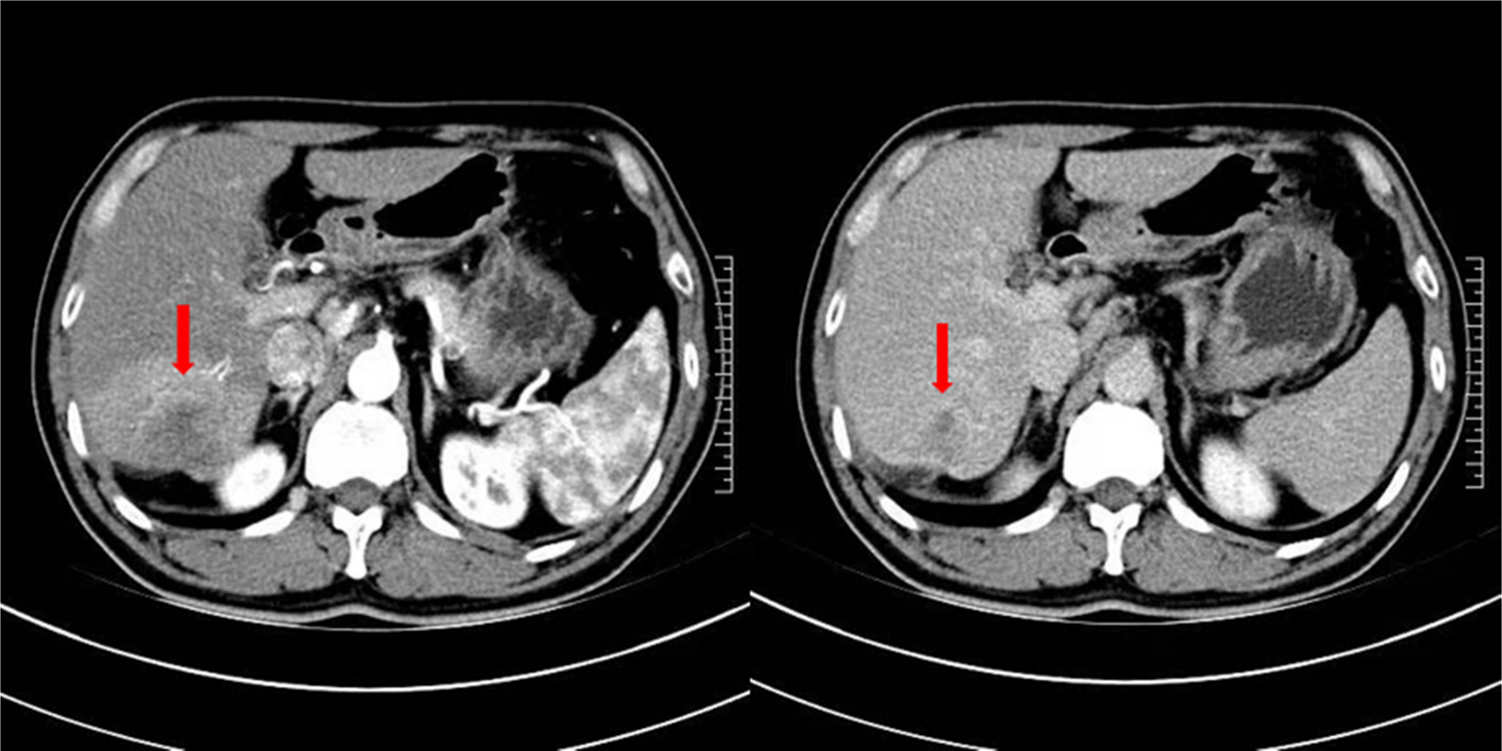
CT-scan showing the size of lesion. The arrow points to a lesion of 5.5 cm × 5.1 cm.

A 48-year-old man with no relevant medical history was admitted to our hospital for acute abdominal pain and fatigue on September 12, 2016. Palpation of the right quadrant of the patient showed tenderness, but the test of Murphy sign was negative. On the second day after admission, the patient underwent computerized tomography (CT) scan, showing a 5.5 cm × 5.1 cm lesion adjacent to the liver capsule (Figure 1). Combined with the results of pathological biopsy, the patient was diagnosed with ICC.

Based on the history, symptoms, signs, and auxiliary examination, the patient was diagnosed with ICC Ⅱ (AJCC-UICC TNM stage: 8) and cachexia (ECOG score: 2). Meanwhile, after multidisciplinary consultation, the surgical treatment was not recommended due to the poor nutritional status. Hence, the interventional treatment was recommended after improving the nutritional status of the patient. Finally, transcatheter arterial chemoembolization (TACE) was performed on the patient on September 18, 2016. Briefly, the catheter was superselectively intubated into the tumor supplying artery through the skin. And the end of the tumor supplying artery was embolized with iodized oil emulsion (10 mL) containing chemotherapy drugs (epirubicin hydrochloride needle 10 mg). The patient had no apparent discomfort after surgery. The patient’s liver function was re-examined after 3 days of treatment, with TB: 11 µmol/L, ALT: 20 U/L, AST: 19 U/L, and ALB: 30 g/L.

**Figure 2:**
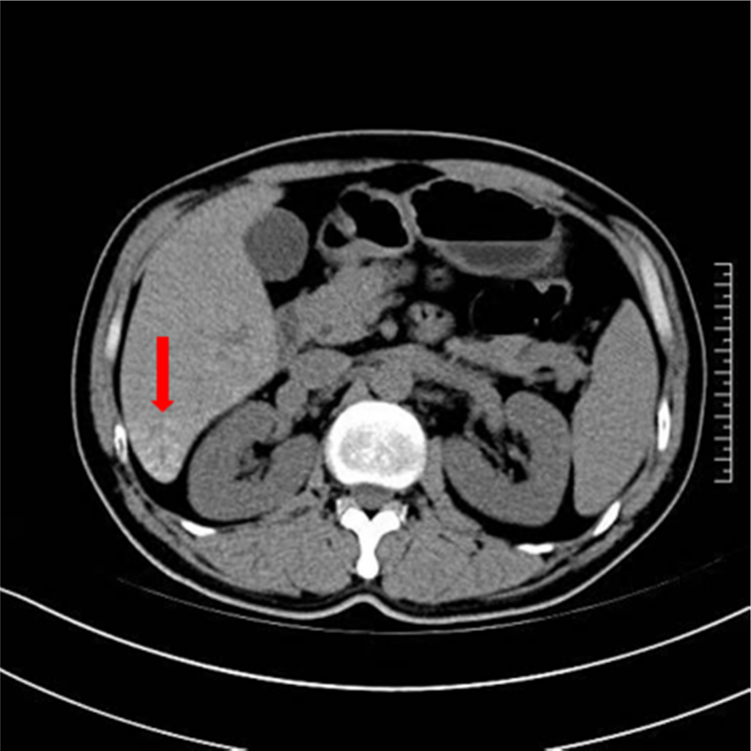
CT-scan showing a little iodized oil deposition shadows.

CT scan of the liver was performed 1 month after TACE treatment. The results showed abnormal right lobe with margin atrophy. The patchy mixed density shadow was observed in the right posterior lobe of liver, and speckled shadow of iodide deposition were observed at the edge (Figure 2). Furthermore, it was assessed as disease stability (SD) by modified Response Evaluation Criteria in Solid Tumor (mRECIST) criteria.

**Figure 3:**
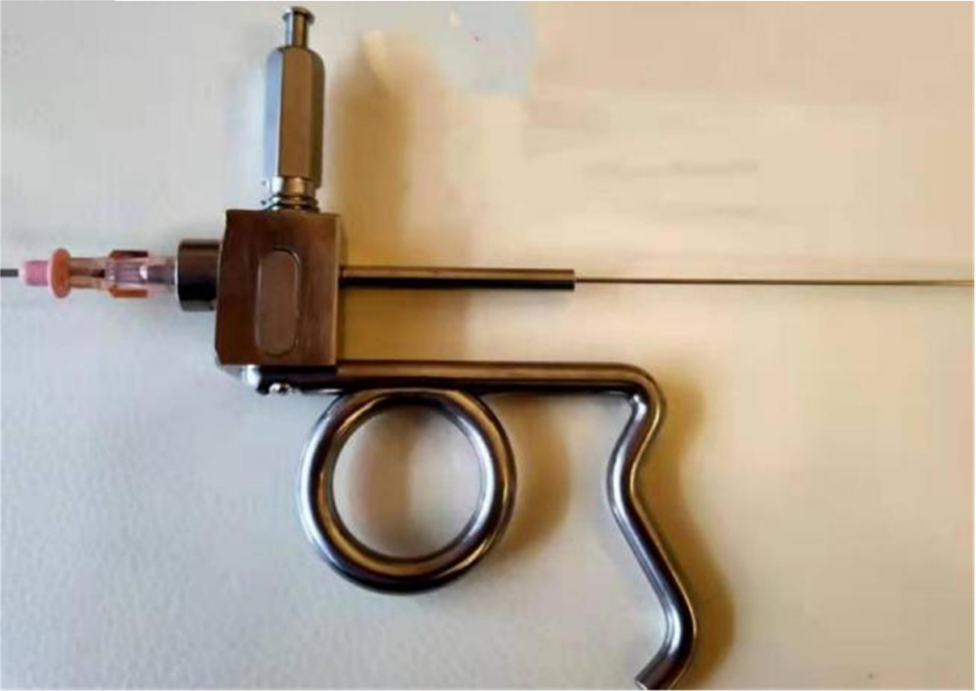
The pistol type particle implantation device.

These results suggested that ICC is not sensitive to TACE treatment. After a multidisciplinary consultation and discussion, the patient underwent minimally interventional therapy with iodine-125 seed implantation. Briefly, based on preoperative CT scan, treatment planning system (TPS; Beijing Hang Kelin Technology Development Co., LTD) was used to simulate approximate dose distribution of solid tumor at prescribed dose and to determine the particle number and spatial distribution (V100 ≥ 95% and D90 ≥ 100%). In order to prevent the particles from entering the blood vessels and migrating, the total radiation dose was 120 Gy, the particle spacing was about 1 cm, and the particles were implanted 1 cm away from the large blood vessels. 3D printing templates were made by TPS before treatment The radioactive particles were placed percutaneously through a 3D-printed template under CT guidance. Complications were observed after operation, including whether there was bleeding under the liver capsule, large blood vessels and whether there was particle displacement or gas embolism in the heart. Moreover, a CT scan was performed after the surgery to make sure the surgery went well. The instruments used in the present operation included pistol type particle implantation device (Figure 3), 18G particle implantation needle, and iodine-125 radioactive particles with an activity of 2.22E + 7 Bq (Beijing Atomic Technology Co., LTD).

**Figure 4:**
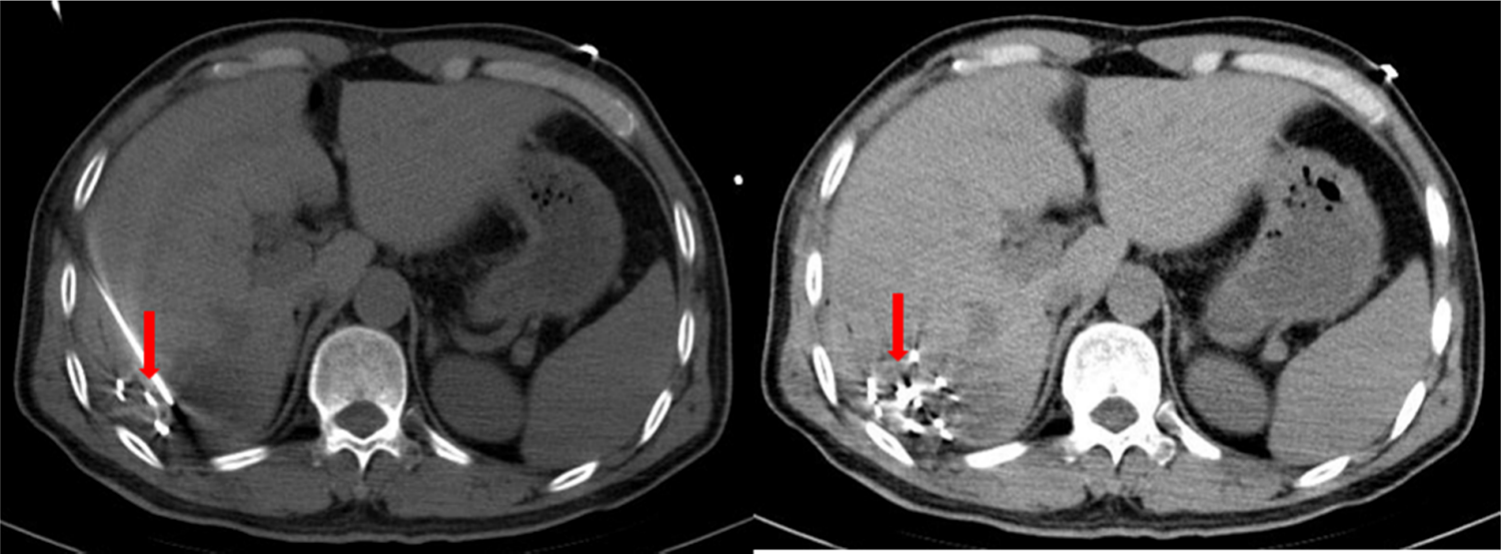
Iodine-125 seed implantation around the lesion in a fan shape with a distance of 1 cm between each two seeds with CT guidance.

In order to avoid bleeding, only two injections were used. We adjusted the needle in the liver for multi-point ablation, always avoiding penetrating the liver capsule. The frame heads were adjusted to fan shape, and iodine-125 seeds were planted so that they were distributed in a fan shape with a distance of 1 cm between each two seeds (Figure 4).

**Figure 5:**
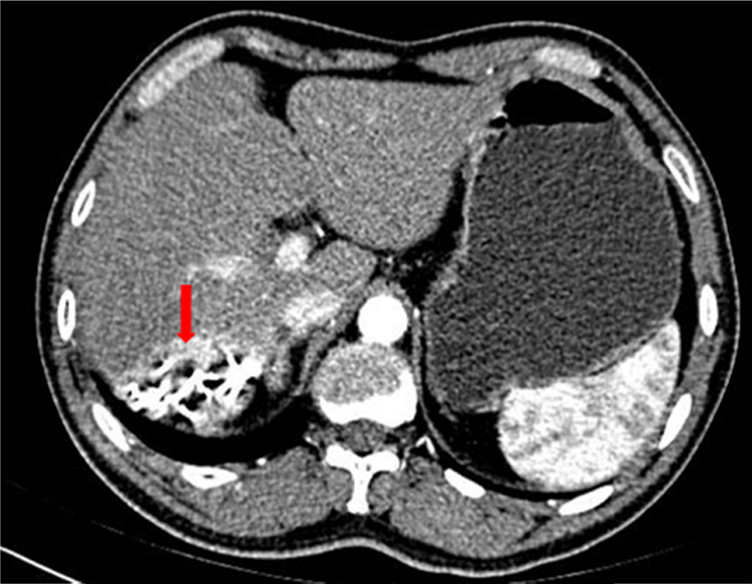
CT-scan showing that lesions were completely necrotic and no signs of recurrence after 3 months of treatment.

**Figure 6:**
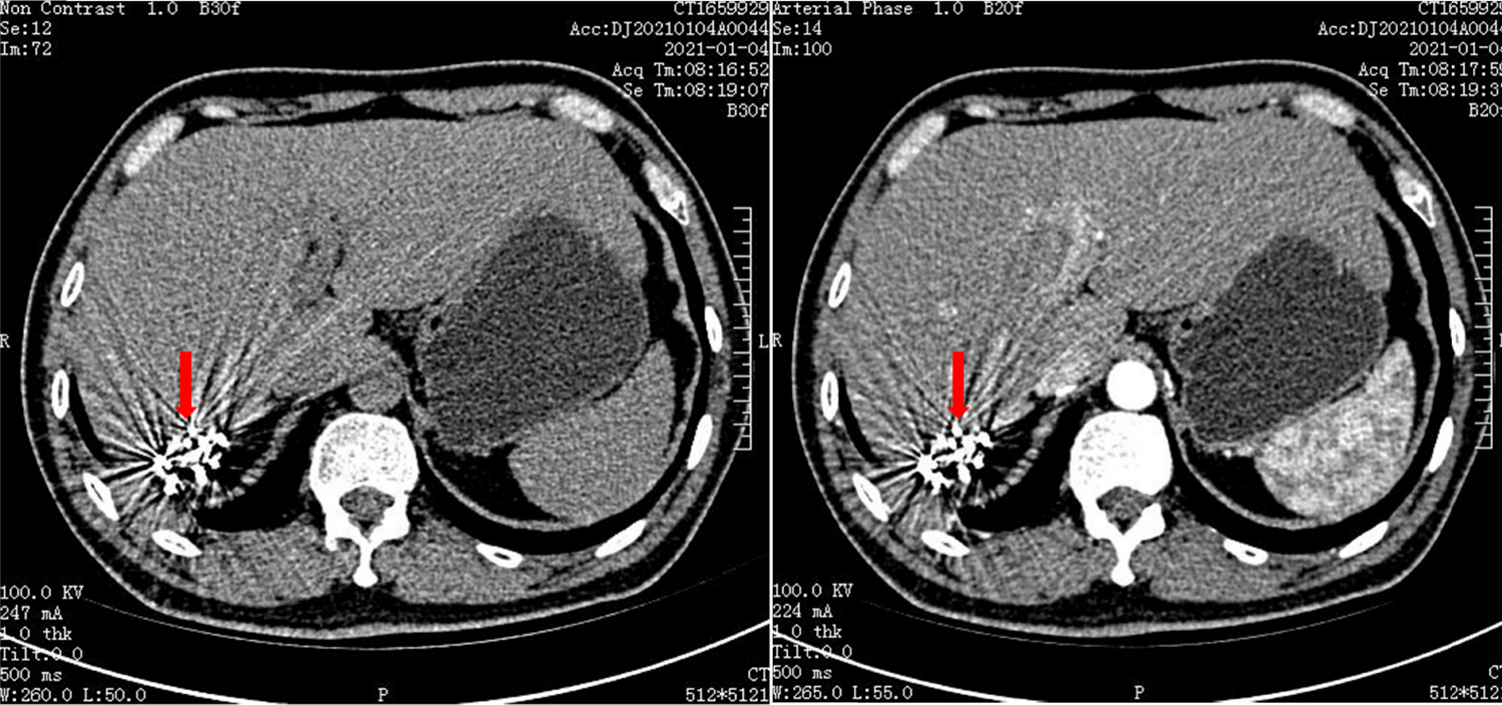
CT-scan showing that lesions were completely necrotic and no signs of recurrence after 5 years of treatment.


After 3 months of treatment, CT scan showed that the lesion was atrophic (Figure 5). The mRECIST assessment showed complete remission (CR). The patient’s liver function was re-examined on January 1, 2021, and the important clinical indicators of liver function results were as follows: TB: 11.2 μmol/L, ALT: 2018 U/L, AST: 16.9 U/L, and ALB: 47 g/L. Moreover, re-examinations after discharge showed that the lesions were completely necrotic and no signs of recurrence until last CT scan on January 1, 2021 (Figure 6), and mRECIST assessment was CR.

**Informed consent:** Informed consent has been obtained from all individuals included in this study.
**Ethical approval:** The research related to human use has been complied with all the relevant national regulations, institutional policies and in accordance with the tenets of the Helsinki Declaration, and has been approved by the ethics committee of The First Affiliated Hospital of Henan University of Chinese Medicine.

## 2 Discussion

Surgical resection is currently the main modality for the treatment of ICC. Due to the insidious onset of ICC, no obvious clinical manifestations, and obvious invasiveness, only 35% of patients are fully eligible for surgery. However, due to the high malignancy of ICC, the postoperative recurrence and metastasis rate is high [12].

The efficacy of TACE in the treatment of ICC is uncertain. Other minimally invasive interventions include radiofrequency ablation and iodine-125 particle implantation. In this case, we found that ICC was not sensitive to TACE treatment, and radiofrequency ablation treatment was too damaging to the patient. After consultation, we treated the patient with iodine-125 seed implantation and the patient completely was cured by minimal interventional therapy with iodine-125 seed implantation.

The main clinical treatment of ICC is surgical resection; however, some types of ICC are not suitable for surgery, or the risks of surgery are too high for the patients. Interventional therapy has become the main treatment for these patients. At present, minimally interventional therapy includes TACE and local treatment (ablation, seed implantation, and external radiotherapy) [13]. However, in the present study, we found that this case with ICC was not sensitive to TACE treatment.

As one of the curative methods for liver cancer, radiofrequency ablation has the advantages of wide application, simple operation, and quick recovery after surgery. However, for tumors in some special parts (such as near the biliary tract, gallbladder, large blood vessels, large tumors protruding from the liver capsule, etc.), it is difficult to cure large tumors (greater than 5 cm) with radiofrequency ablation. Actually, radiofrequency ablation is suitable for tumors smaller than 3 cm. In this study, the lesions of this patient were non-encapsulated and had obvious edge enhancement, indicating that the lesions were infiltrative growth and the surrounding tumor cells were actively growing. If radiofrequency ablation is performed, the ablation range needs to be expanded, at least 1–2 cm beyond the periphery to ensure complete inactivation of the tumor, which will cause greater damage.

During the last few decades, several brachytherapy methods for ICC have been reported. For example, 90Y radioembolization therapy, a form of brachytherapy, has been regarded as a locoregional treatment in unresectable ICC patients [14,15,16]. Iodine-125 seed implantation is a method of internal radiotherapy to treat tumors [17]. It targets tumor cell by continuously releasing low-dose gamma rays at close distances but does less damage to other tissues around the tumor [18,19]. Accumulating evidence reveals that iodine-125 seed implantation could be a safe and effective treatment for advanced extrahepatic cholangiocarcinoma [11]. In this case, we used minimally interventional therapy with iodine-125 seed implantation. Iodine-125 particles can slowly release gamma rays, which can directly destroy tumor tissue. The radiation range of each particle is 17 mm, and the half-life is 59.43 days. According to the inverse square law of tissue dose distance, the radiation dose outside the planned area is gradually attenuated, so that the target lesion and the surrounding 1–2 cm tissue can be killed to the maximum extent, which has the effect of similar enlarged ablation, and avoids the “thermal deposition” of adjacent blood vessels during radiofrequency ablation “effect.” Moreover, it does little damage to the surrounding tissue, and is especially suitable for tumors in special parts.

In this case, the tumor was large and had invaded the liver capsule and intrahepatic blood vessels. The physical fitness score showed that the patient was not suitable for surgery, and radiofrequency ablation was difficult to achieve a one-time radical cure. Therefore, iodine-125 seed implantation was selected. However, it is very difficult to make the iodine-125 seed evenly distributed around the lesion. We introduced in detail the operation details and matters needing attention. During the execution of the iodine-125 seed implantation operation, we adjusted the frame head to make it fan-shaped, and implanted iodine-125 seed around the lesion. After iodine-125 implantation treatment, the patient has survived tumor-free for more than 4 years, which proves that iodine-125 particles have obvious effects in the treatment of ICC. Our team has accumulated some experience in the application of iodine-125 in the treatment of hepatocellular carcinoma. We have analyzed 77 cases (including 33 cases of seed implantation and 44 cases of radiofrequency ablation) with solitary tumors less than 5 cm in the liver. The local recurrence rates of the two groups of patients at 1, 2, and 3 years were compared. The results showed that there was no significant difference in the recurrence rate between the seed implantation group and the radiofrequency ablation group in terms of controlling the local recurrence of small hepatocellular carcinoma [20]. However, iodine-125 particles have been less reported in the treatment of larger ICC. This interventional approach needs to be validated in more future experiments. This case provides an important reference for the selection of ICC treatment options.

## 3 Conclusion

Collectively, our case provides successful experience and new inspiration for the treatment of ICC. Therefore, interventional therapy with iodine-125 seed implantation may be a promising therapeutic strategy for ICC.
